# Effect of bone marrow-derived mesenchymal stem cells on hepatic fibrosis in a thioacetamide-induced cirrhotic rat model

**DOI:** 10.1186/s12876-014-0198-6

**Published:** 2014-11-25

**Authors:** Yoon Ok Jang, Moon Young Kim, Mee Yon Cho, Soon Koo Baik, Youn Zoo Cho, Sang Ok Kwon

**Affiliations:** Department of Internal Medicine, Yonsei University, Wonju College of Medicine, 162, Ilsan-dong, Wonju, Republic of Korea; Department of Pathology, Yonsei University, Wonju College of Medicine, 162, Ilsan-dong, Wonju, Republic of Korea

**Keywords:** Bone marrow-derived mesenchymal stem cell, Cirrhosis, Hepatic fibrosis, Liver function

## Abstract

**Background:**

Cirrhosis is a long-term consequence of chronic hepatic injury with fibrosis. No effective therapy is currently available for decompensated cirrhosis except liver transplantation. Hence, we investigated the effect of bone marrow-derived mesenchymal stem cells (BM-MSCs) on hepatic fibrosis in a thioacetamide (TAA)-induced cirrhotic rat model.

**Methods:**

The BM-MSCs were injected directly into the right liver lobe twice, at 6 and 8 weeks during the 12-week TAA administration, in thioacetamide (TAA)-induced cirrhotic rats model, and hepatic fibrosis was evaluated. At 12 weeks, the effect of BM-MSCs on hepatic fibrosis was analyzed histomorphologically using the Laennec fibrosis scoring system, and the collagen proportionate area was quantified. Cirrhosis-related factors, such as transforming growth factor β1 (TGF-β1), type 1 collagen (collagen-1), α-smooth muscle actin (α-SMA), and P-Smad3/Smad3 expression levels, were evaluated using real-time polymerase chain reaction and western blot assays.

**Results:**

According to the Laennec fibrosis scoring system, histological improvement was observed in hepatic fibrosis after BM-MSC treatment (*P* <0.01). The percentage of the collagen proportionate area decreased from 16.72 ± 5.51 to 5.06 ± 1.27 after BM-MSC treatment (*P* <0.01). The content of hepatic hydroxyproline was significantly lower in the BM-MSC treated group (46.25 ± 13.19) compared to the untreated cirrhotic group (85.81 ± 17.62; *P* <0.01). BM-MSC administration significantly decreased TGF-β1, collagen-1, and α-SMA expression in TAA-induced cirrhotic rats (*P <*0.01). We also confirmed P-Smad3/Smad3, downstream effectors of the TGF-β1 signaling pathway, and found that MSC transplantation inhibited Smad3 phosphorylation.

**Conclusions:**

BM-MSC treatment attenuated hepatic fibrosis in rats with TAA-induced cirrhosis, raising the possibility of the clinical use of BM-MSCs in the treatment of cirrhosis.

**Electronic supplementary material:**

The online version of this article (doi:10.1186/s12876-014-0198-6) contains supplementary material, which is available to authorized users.

## Background

Cirrhosis, the end stage of progressive hepatic fibrosis, is characterized by distortion of the hepatic architecture and the formation of regenerative nodules, angiogenesis, and shunts, leading to loss of liver function and the development of hepatocellular carcinoma [1–6]. The most effective therapy for advanced cirrhosis is currently liver transplantation. However, this procedure has several limitations, including a lack of donors, surgical complications, immunological suppression, and a high medical cost [7]; thus, there is a need for a new therapeutic paradigm in this field.

Cell-based therapy, such as hepatocyte transplantation, has been considered a potential alternative to liver transplantation. Alternative therapeutic approaches that circumvent the use of the whole organ, such as the transplantation of cells of various origins, have recently been accepted [8,9]. In addition, stem cell transplantation has been suggested as an effective alternate therapy for hepatic disease [10,11]. Several previous studies using animal models of liver diseases have demonstrated that bone marrow (BM) cell transplantation may accelerate the liver regeneration process, reduce hepatic fibrosis, and improve liver function and survival [12–15]. The prospects for stem cell transplantation as a therapy for hepatic disease, as determined by initial translational pilot studies testing the direct hepatic administration of BM-derived stem cells, have been encouraging and have suggested enhanced liver regeneration prior to partial hepatectomy and improved liver function in advanced chronic liver disease [16–22]. Among the stem cells, mesenchymal stem cells (MSCs) in particular have practical advantages in regenerative medicine due to their high capability for self-renewal, their potential for multipotent differentiation, and their low immunogenicity. In addition, previous studies have demonstrated that bone marrow-derived mesenchymal stem cells (BM-MSCs) might be involved in the regression of liver fibrosis [23,24].

Concomitantly, transforming growth factor (TGF)-β1 is a key mediator of fibrogenesis [25], and the TGF-β1 signaling pathway contributes to liver fibrosis progression [26]. More importantly, TGF-β1 mediates its biological functions via the canonical Smad pathway by activating the transmembrane receptors that stimulate the cytoplasmic Smad proteins, which in turn activate collagen transcription [26,27]. Therefore, the TGF-β1-activated Smad3 signaling pathway is critical for the development of hepatic fibrosis, and TGF-β signaling pathways are potential therapeutic targets for liver fibrosis [27–29].

BM-MSCs can be easily harvested from bone marrow, expanded ex vivo, and differentiated into many cell type lineages, if desired. Because of their immunotolerance, the establishment of MSCs as effective universal donor cells [30] could then dramatically expand their therapeutic potential for cellular cardiomyoplasty. Despite the initial hope that BM-MSCs could feature an immune privilege, it is now increasingly recognized that these cells trigger an immune reaction that leads to their rejection in both allogeneic and xenogeneic settings. In fact, a number of laboratories have recently reported that MSCs may have a unique immunological property capable of inducing tolerance in immunocompetent allotransplant or even xenotransplant recipients [31]. The mechanisms of such immunotolerance have been the subject of intense study, and three interrelated candidate mechanisms are emerging [30,32]. MSCs appear to evade rejection by being hypoimmunogenic, modulating T-cell phenotypes, and immunosuppressing the local environment.

In this study, we investigated the effect of BM-MSCs on hepatic fibrosis in a thioacetamide (TAA)-induced cirrhotic rat model and the underlying mechanism by which BM-MSCs ameliorate hepatic fibrosis.

## Methods

### Preparation of human bone marrow-derived mesenchymal stem cells

Human BM-MSCs were obtained from healthy persons who voluntarily donated their bone marrow stem cells. Approximately 10–20 mL of BM was aspirated from the posterior iliac crests of humans under local anesthesia. BM mononuclear cells were isolated through density-gradient centrifugation (Histopaque-1077, Sigma-Aldrich, St. Louis, MO, USA). Mononuclear cells (2–3 × 10^5^ cells/cm^2^) were plated in a 75-cm^2^ flask (Falcon, Franklin Lakes, NJ, USA) with Dulbecco’s modified Eagle’s medium (DMEM; Gibco, Grand Island, NY, USA) containing 10% fetal bovine serum (FBS; Gibco) and 1% penicillin/streptomycin (Gibco) and then cultured at 37°C in a 5% CO_2_ atmosphere. When the cultures approached 80% confluence, the cells were harvested by treatment with a trypsin/EDTA solution (Gibco) and replated at a density of 4–5 × 10^3^ cells/cm^2^ in 175-cm^2^ flasks. Cells for injection were serially subcultured to passage four or five. The human procedures and protocols were approved by the Institutional Review Board (IRB) at Yonsei University Wonju Severance Hospital (CR109021), and conducted according to the principles of the Declaration of Helsinki. All participants provided written informed consent prior to participation in the study.

### Immunophenotypes and differentiation assays of BM-MSCs

**Figure 1:**
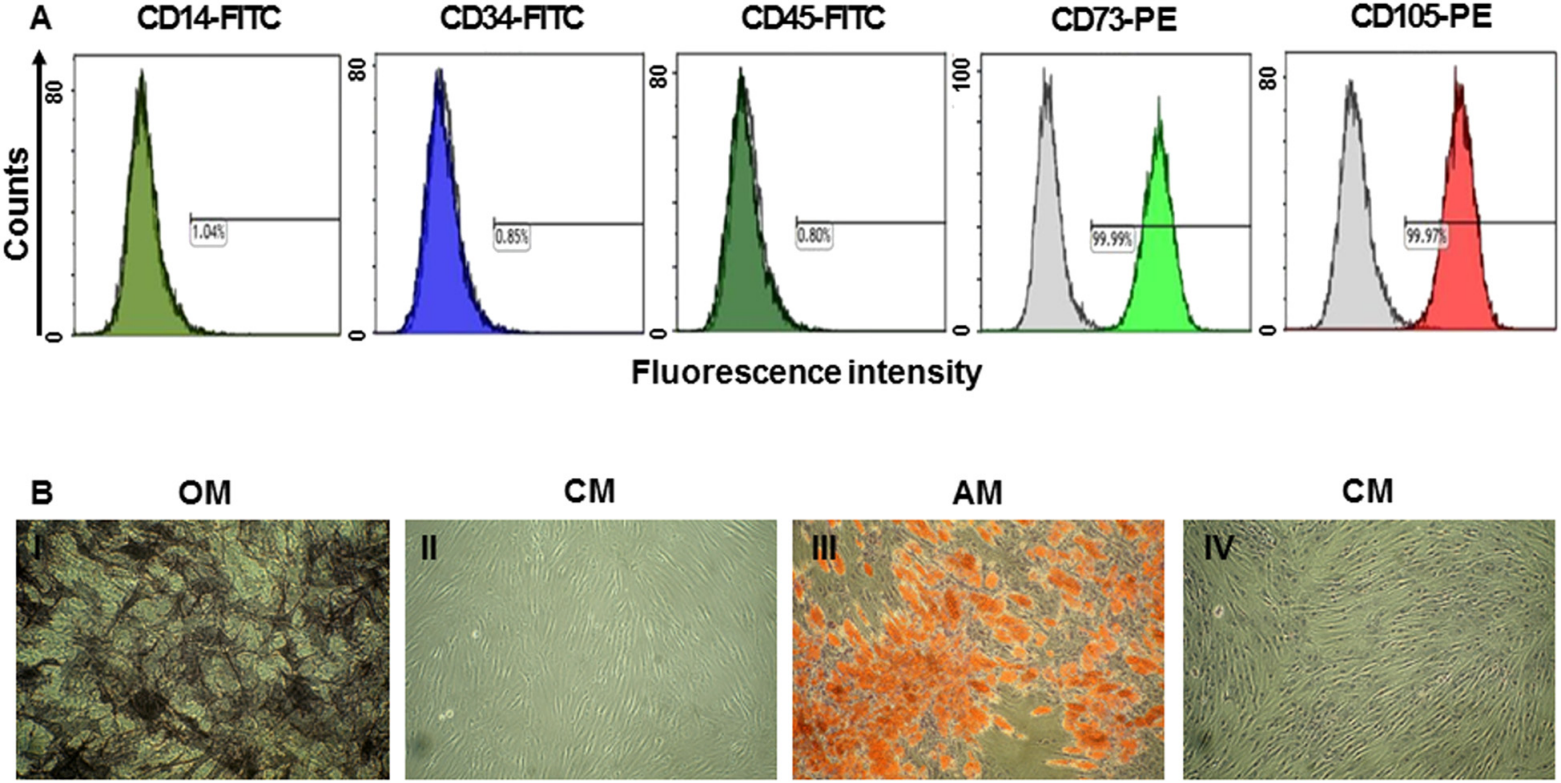
**Immunophenotypes and differentiation potentials of the BM-MSCs. (A)** The expressions of cell-surface antigens (CD14, CD34, CD45, CD73, and CD105) were evaluated through flow cytometry. **(B)** BM-MSCs stained positively for endogenous alkaline phosphatase activity, indicating osteogenic differentiation within an osteogenic medium (OM; I), or stained negatively in control medium (CM; II). BM-MSCs stained positively for lipid droplets, indicating adipogenic differentiation within adipogenic medium (AM; III), or stained negatively in CM (IV) (Magnifications × 100).

The immunophenotypes of the BM-MSCs (CD14, CD34, CD45, CD73, and CD105) were analyzed on the day of injection, and their differentiation potentials were determined (osteogenesis and adipogenesis; Figure 1). For the immunophenotype analysis, BM-MSCs were stained with antibodies conjugated with fluorescein isothiocyanate (FITC) or phycoerythrin (PE): CD14-FITC, CD34-FITC, CD45-FITC, CD73-PE, and CD105-PE (BD Biosciences, San Jose, CA, USA). Briefly, 5 × 10^5^ cells were resuspended in 0.2 mL of phosphate-buffered saline (PBS) and incubated with FITC- or PE-conjugated antibodies for 20 min at room temperature. FITC- or PE-conjugated mouse IgGs were used as the control isotype at the same concentration as the specific primary antibodies. The fluorescence intensity of the cells was evaluated through flow cytometry (Epics XL; Beckman Coulter, Miami, FL, USA). Osteogenic differentiation was determined by first plating the cells at 2 × 10^4^ cells/cm^2^ in six-well plates and then incubating them in the following osteogenic medium for 2–3 weeks: DMEM medium supplemented with 10% FBS, 10 mM β-glycerophosphate, 10^−7^ M dexamethasone, and 0.2 mM ascorbic acid (Sigma-Aldrich) [33]. The osteogenic differentiation was quantified from the release of p-nitrophenol from p-nitrophenyl phosphate by the enzyme alkaline phosphatase [34]. For adipogenic differentiation, BM-MSCs were plated at 2 × 10^4^ cells/cm^2^ in six-well plates and cultured for 1 week; then, differentiation was induced with adipogenic medium (10% FBS, 1 μM dexamethasone, 0.5 mM 3-isobutyl-1-methylxanthine, 10 μg/mL insulin, and 100 μM indomethacin in high-glucose DMEM) for an additional 3 weeks. The differentiated cells were fixed in 4% paraformaldehyde for 10 min and stained with fresh Oil Red-O solution (Sigma-Aldrich) to display lipid droplets (Figure 1B). The criteria regarding the use of MSCs included viability greater than 80%; the absence of microbial contamination (bacteria, fungus, virus, or mycoplasma) when tested 3–4 days before administration; CD73 and CD105 expression in more than 90% of the cells; and the absence of CD14, CD34, and CD45 in less than 3% of the cells, as assessed by flow cytometry.

### Induction of hepatic fibrosis and BM-MSC treatment in a thioacetamide-induced cirrhotic rat model

**Figure 2:**
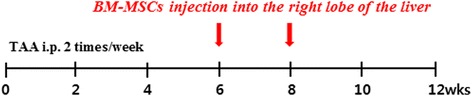
**Experimental design of BM-MSCs injection.** Hepatic fibrosis was induced in Sprague–Dawley rats by intraperitoneal (i.p.) injections of TAA (300 mg/kg) twice a week for 12 weeks. The BM-MSCs were injected directly into the right liver lobe twice, at 6 and 8 weeks during the 12-week TAA administration.

Seven-week-old male Sprague–Dawley rats (Orient Bio Inc.) were maintained at a room temperature of 25°C with a 12/12-h light/dark cycle and with free access to food and water throughout the 12-week experiment. Hepatic fibrosis was induced in Sprague–Dawley rats by intraperitoneal (i.p.) injections of TAA (Sigma, St. Louis, MO, USA; 300 mg/kg body weight) twice a week for 12 weeks. The animals were randomly allocated into three groups (each group, *n* =18) as follows: Group I (G1, sham group); Group II (G2, untreated cirrhotic group), which received the TAA injection; and Group III (G3, BM-MSC treated group), which received the TAA injection and the BM-MSC treatment. The rats were anesthetized by intramuscular administration with a mixture of Zoletil® (Virbac Laboratories, Carros, France) and Rompun® (Bayer Korea, Seoul, Korea). With aseptic techniques, a 1-cm incision was made caudal to the costal arch on the right flank to expose the right lobe of the liver. With a syringe, 2 × 10^6^ human BM-MSCs were injected directly into the right lobe of the liver at 6 and 8 weeks during the 12-week TAA administration in the BM-MSC treated group (Figure 2). At 12 weeks, the animals were sacrificed after taking blood samples, and liver tissue specimens were collected, fixed, and immediately snap-frozen and stored at −80°C for analysis. The animal experimental procedures and protocols were approved by the Institutional Animal Care and Use Committee (IACUC) at Yonsei University Wonju College of Medicine (YWC-131008-1).

### Histomorphological and immunohistochemical analysis

Five-micrometer-thick sections of paraffin-embedded liver tissues were prepared and stained with hematoxylin and eosin (H&E), Masson’s trichrome (MTC), α-smooth muscle actin (α-SMA), and Picrosirius Red. Fibrosis was evaluated using the Laennec fibrosis scoring system (Table 1). In the Laennec system, the thickness of the predominant type of septae in each specimen is chosen, and the smallest nodule is selected for scoring. A liver pathologist who was blinded to the clinical data evaluated fibrosis simultaneously and prospectively. The Laennec fibrosis scoring system was used because this system divides cirrhosis into three subclasses, allowing for a more detailed estimation of fibrosis after intervention [35]. In addition, to estimate any treatment-induced changes in liver fibrosis, the fibrosis area was quantified as a percentage of the total area that was positive for MTC stain in the digital photomicrographs using a computerized image analysis system (Analysis 3.0, Soft Imaging System, Münster, Germany). To quantify the fibrosis area, microscopic areas were selected randomly at an original magnification of 100X. For immunohistochemical analysis, the tissue sections were incubated with primary antibody against α-SMA (diluted 1:800; Neomarkers, Fremont, CA, USA) for 90 min at room temperature after washing with buffer. The tissue sections were incubated with the chromogen 3-amino-9-ethylcarbazole (BioGenex, San Ramon, CA, USA) for 5–7 min. Prior to mounting, the sections were counterstained with hematoxylin and then dehydrated. An UltraVision LP Large Volume Detection System (Lab Vision, Runcorn, UK) was used as the detection system. Morphological analysis of immunopositive cells was also performed using a computerized image analysis system.

**Table 1:** **Laennec scoring system for staging fibrosis in liver specimens**

**Stage**	**Name**	**Septa (thickness and number)**	**Criteria**	**Score**
0	No definite fibrosis			0
1	Minimal fibrosis	+/−	No septa or rare thin septum; may have portal expansion or mild sinusoidal fibrosis	1
2	Mild fibrosis	+	Occasional thin septa; may have portal expansion or mild sinusoidal fibrosis	2
3	Moderate fibrosis	++	Moderate thin septa; up to incomplete cirrhosis	3
4A	Mild, definite, or probable cirrhosis	+++	Marked septation with rounded contours or visible nodules. Most septa are thin (one broad septum allowed)	4
4B	Moderate cirrhosis	++++	At least two broad septa, but not very broad septa and less than half of biopsy length composed of minute nodules	5
4C	Severe cirrhosis	+++++	At least one very broad septum or more than half of biopsy length composed of minute nodules (micronodular cirrhosis)	6

Picrosirius Red staining was performed to quantify the total amount of collagen. Five-micrometer-thick sections of paraffin-embedded liver tissues were deparaffinized and rehydrated with distilled water and stained with a Picrosirius Red staining kit (Polysciences, Warrington, PA, USA) according to the manufacturer’s instructions. In addition, the amount of collagen (the main component of fibrous tissue) was estimated from the collagen proportionate area and expressed as the percentage of the total area that was positive for Picrosirius Red stain on microscopy (Olympus BX51, Tokyo, Japan) using a computerized image analysis program (IMT i solution, Vancouver, Canada). While measuring the collagen proportionate area, we eliminated image artifacts and structural collagen in large portal tracts and blood vessel walls [36].

### Hepatocyte differentiation and identification of BM-MSCs

The expression of hepatocyte specific marker (Albumin) was analyzed at 7 and 14 days after 2 × 10^6^ human BM-MSCs were injected directly into the right lobe of the liver in TAA-induced hepatic fibrosis rats. Five-micrometer-thick sections of liver tissues were incubated with human anti-albumin antibody (A6684; Sigma) at 4°C overnight. The slides were washed and incubated with Alexa Fluor® 488 (Invitrogen, Carlsbad, CA) secondary antibody for 1 hr in dark. The nuclei were stained with Hoechst. Fluorescence images were showed under a laser scanning confocal microscope (TCS SPE, Leica Microsystems GmbH, Wetzlar, Germany). To identify injected BM-MSCs in the hepatic fibrosis rat liver, BM-MSCs were labeled with fluorescent spherical silica nanoparticles with CELL-STALKER™-CSR (Biterials) that contained Rhodamine B isothiocyanate (RITC) according to manufacturer’s protocol. CELL-STALKER was centrifuged at 12,000 rpm for 10 min then supernatant was discarded except pellet and sonicated for 5 min at 40 khz-300 w to evenly distribute particles, placed into BM-MSCs culturing 75 T flask and incubated at 37°C for 24 hrs. After incubation, the cells were harvested by treatment with a trypsin/EDTA solution. The 2 × 10^6^ BM-MSCs labeled with CELL-STALKER were injected directly into the right lobe of the liver in TAA-induced hepatic fibrosis rats and fluorescence images for BM-MSCs were showed under a laser scanning confocal microscope at 0, 3, 7, 14 days.

### Biochemical analysis

Measurement of alanine transaminase (ALT), aspartate transaminase (AST), total bilirubin, and albumin levels were carried out using commercially available kits (Asan Pharmaceutical, Republic of Korea) according to manufacturer’s instructions.

### Quantitative real-time PCR analysis

Total RNA was isolated from liver tissues using TRIzol reagent (Invitrogen, Carlsbad, CA, USA) according to the manufacturer’s protocol. RNA purity and concentration were determined using a spectrophotometer (Ultrospec 2100 pro UV/Visible, Amersham Bioscience, Freiburg, Germany). cDNA was synthesized from total RNA (1 μg) using the GeneAmp RNA PCR Kit (Applied Biosystems, Foster City, CA, USA) with oligo-dT (Applied Biosystems). For the real-time polymerase chain reaction (PCR), amplification was performed to measure the mRNA levels of TGF-β1, type 1 collagen (collagen-1), and α-SMA using sequence-specific primers (Table 2). Quantitative real-time PCR using SYBR Green PCR Master Mix (Applied Biosystems) was performed in an ABI PRISM 7900HT Sequence Detection System (Applied Biosystems) according to the manufacturer’s instructions. The data were analyzed using SDS 2.2.2 software (Applied Biosystems). The cycle threshold (Ct) values of the target genes were normalized to the Ct values of the endogenous control (glyceraldehyde-3-phosphate dehydrogenase). Relative changes were calculated using the equation 2^–ΔΔCt^.

**Table 2:** **Primer sequences for quantitative PCR**

**Gene**	**Forward/reverse**	**Primer sequence**
GAPDH	Forward	AGCCCAGAACATCATCCCTG
	Reverse	CACCACCTTCTTGATGTCATC
TGF-β1	Forward	CCTGGAAAGGGCTCAACAC
	Reverse	CAGTTCTTCTCTGTGGAGCTGA
Collagen-1	Forward	CATGTTCAGCTTTGTGGACCT
	Reverse	GCAGCTGACTTCAGGGATGT
α-SMA	Forward	CGATAGAACACGGCATCATCAC
	Reverse	GCATAGCCCTCATAGATAGGCA

GAPDH, glyceraldehyde-3-phosphate dehydrogenase; TGF-β1, transforming growth factor-beta 1; collagen-1, type 1 collagen; α-SMA, α-smooth muscle actin.

### Hepatic hydroxyproline content

Liver tissues were hydrolyzed with 6 N HCl at 120°C for 16 hrs. The hydrolysate was then cooled, neutralized with 6 N NaOH, and centrifuged at 13,000 *g* for 10 min. The supernatants were supplemented with 7% chloramine T, acetate/citrate buffer (sodium acetate·3H_2_O, trisodium citrate·2H_2_O, citric acid, with isopropanol). Ehrlich’s solution (dimethylaminobenzaldehyde with perchloric acid and isopropanol) was then added and incubated at 60°C for 35min. After cooling, absorbance was measured at 560 nm with an Emax Precision Microplate Reader (Molecular Devices, Sunnyvale, CA, USA). Hydroxyproline concentration was calculated from a standard curve prepared with hydroxyproline (Sigma, H5534). The results were expressed as micrograms of hydroxyproline per gram of liver tissue.

### Western blot assays

For total protein extracted from liver tissues, tissues were homogenized using a TissueLyser II (QIAGEN GmbH, Haan, Germany) with a tissue protein extraction reagent (T-PER; Pierce, Rockford, IL, USA). The lysates were centrifuged at 13,000 rpm for 15 min at 4°C, and the protein concentrations of the supernatants were determined using a protein assay kit (Bio-Rad Laboratories Inc., Hercules, CA). Thirty micrograms of each liver protein was electrophoresed on a 10% sodium dodecyl sulfate-polyacrylamide (SDS-PAGE) gel and then transferred to polyvinylidene difluoride (PVDF) membranes (Millipore, Bedford, MA, USA). The membranes were blocked with 5% skim milk in Tris-Buffered Saline (TBS) containing 0.1% Tween-20 for 1 hr at room temperature, and the membranes were then incubated with primary antibodies at 4°C overnight. The primary antibodies were as follows: TGF-ß1 (Abcam, Cambridge, MA, USA), α-SMA (Abcam), Smad3 (Cell Signaling Technology, Danvers, MA, USA), and phospho Smad3 (Cell Signaling Technology). Horseradish peroxidase (HRP)-conjugated secondary antibodies against either mouse IgG (Abcam) or rabbit IgG (Cell Signaling Technology) were incubated for 1 hr at room temperature. Specific protein bands on the membranes were developed using an enhanced chemiluminescence (ECL) detection kit (Amersham, Piscataway, NJ, USA). To normalize between experiments, the membranes were probed with a β-actin antibody (Abcam), and the intensity of each protein band was normalized to the intensity of β-actin.

### Statistical analysis

The values are expressed as means ± standard deviations. Nonparametric analysis was performed with the Kruskal-Wallis *H* test. Statistical analysis was performed using SPSS software version 20.0 (SPSS, Chicago, IL, USA). For all tests, *P* values <0.01 were considered significant.

## Results

### Immunophenotypes and differentiation potentials of the BM-MSCs

The immunophenotypes for CD14, CD34, CD45, CD73, and CD105 cells were determined, and osteogenic or adipogenic differentiation was induced on the day of BM-MSC injection (Figure 1). In both the first and second injected cell populations, CD73 or CD105 (which are positive markers of BM-MSCs) were expressed in more than 98% of the cells. However, CD14, CD34, and CD45 (which are known to be negative markers of BM-MSCs) were expressed in less than 1% of the cells (Figure 1). Therefore, the BM-MSCs were differentiated into osteocytes and adipocytes (Figure 1B).

### Histological and immunohistochemical analysis

**Figure 3:**
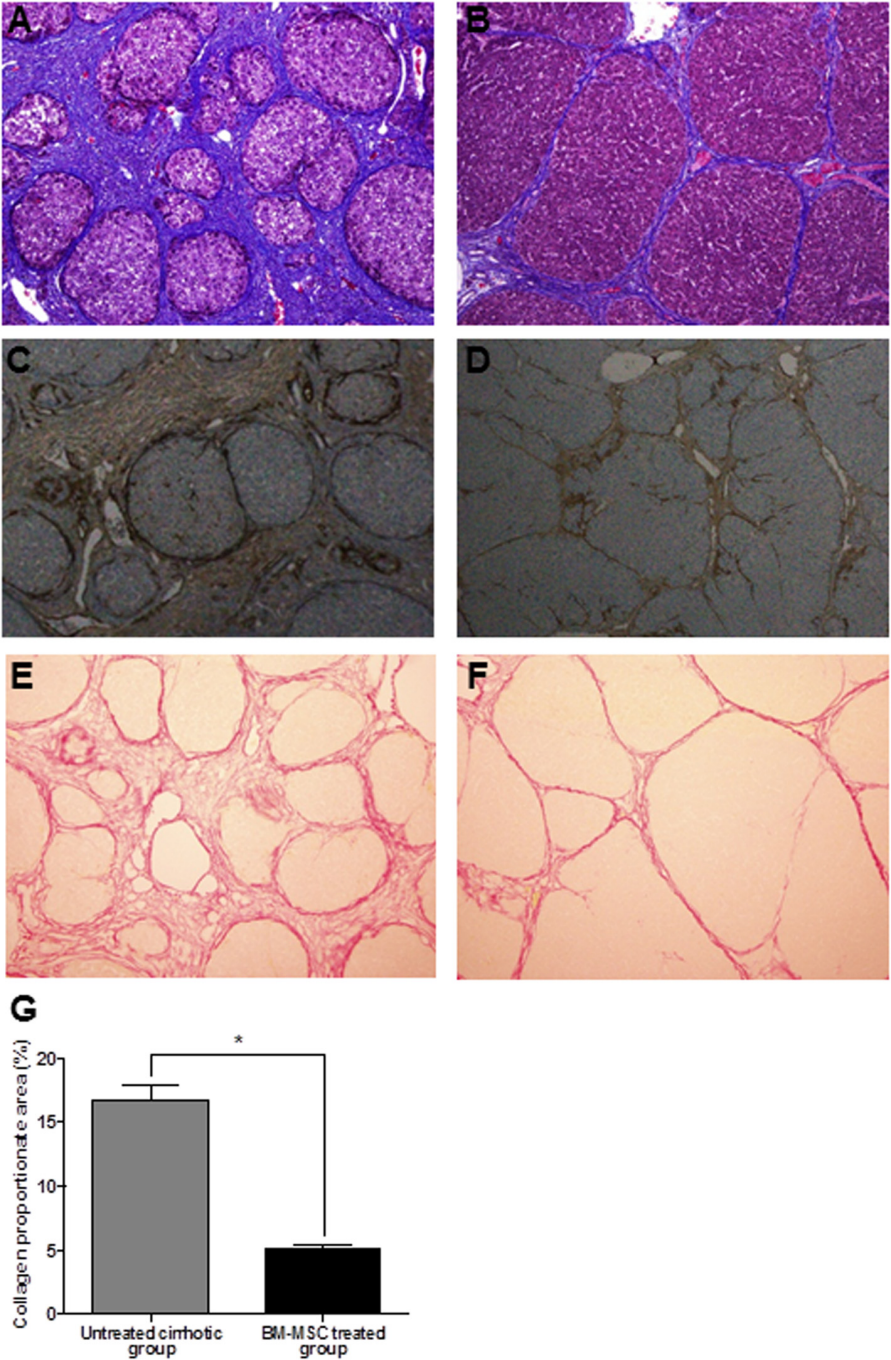
**Histological analysis and the relative expression of the collagen proportionate area.** Histological analysis was evaluated by MTC **(A, B)**, α-SMA **(C, D)**, and Picrosirius Red staining **(E, F)**. BM-MSC treatment induced an improvement of cirrhosis from F4C **(A, C, E)** to F4A **(B, D, F)** according to the Laennec fibrosis scoring system. Untreated cirrhotic group **(A, C)** show cirrhosis with at least one broad septum with minute nodules (F4C) and BM-MSC treated group **(B, D)** show cirrhosis with marked septation with rounded contours or visible large nodules (F4A). Picrosirius Red staining of a section from a liver biopsy specimen showed a change in collagen proportion stained as red from untreated cirrhotic group **(E)** to BM-MSC treated group **(F)**. The relative area of collagen stained by Picrosirius Red was analyzed with an image analysis program **(G)**. The percentage of the collagen proportionate area decreased from 16.72 ± 5.51 to 5.06 ± 1.27 after BM-MSC treatment (*P <*0.01). (Magnifications × 100). Values are expressed as means ± SD. *P <0.01.

The Laennec fibrosis scoring system revealed detailed individual changes within the cirrhotic tissue (F4A–F4C; Table 1). Histological analysis was evaluated by H&E and MTC staining (Figure 3). In the untreated cirrhotic group, the liver sections were strongly stained by H&E and MTC, showing definite cirrhosis (stage 4 fibrosis) with regenerating nodules and fibrous septae (Figure 3A), whereas the BM-MSC treated group showed mild fibrosis (Figure 3B). To compare these findings, the degree of fibrosis was scored according to the Laennec fibrosis scoring system. The BM-MSC treated group had a significantly lower mean score than that of the untreated cirrhotic group. According to the Laennec fibrosis system, histologically, the BM-MSC treated group showed significant improvement in hepatic fibrosis compared to the untreated cirrhotic group (*P* <0.01). These results were further confirmed by immunohistochemical staining revealing α-SMA expression and Picrosirius Red stain (Figure 3). The relative expression of the collagen proportionate area stained by Picrosirius Red was analyzed using an image analysis program. The percentage of the collagen proportionate area significantly decreased from 16.72 ± 5.51 to 5.06 ± 1.27 after BM-MSC treatment (*P <*0.01; Figure 3G). In addition, the analysis of histopathological fibrosis scoring confirmed that cirrhosis was significantly reduced by BM-MSCs treatment, compared to the untreated cirrhotic group (Table 3).

**Table 3:** **Histological stage of hepatic fibrosis**

**Group**	**Stage 0**	**Stage 1**	**Stage 2**	**Stage 3**	**Stage 4A**	**Stage 4B**	**Stage 4C**	**Average**
Score	0	1	2	3	4	5	6	
G1	18							0
G2					2	7	9	5.4
G3			2	7	6	3		3.6

Group I (G1, sham group); Group II (G2, untreated cirrhotic group); Group III (G3, BM-MSC treated group).

### Hepatocyte differentiation and identification of BM-MSCs

**Figure 4:**
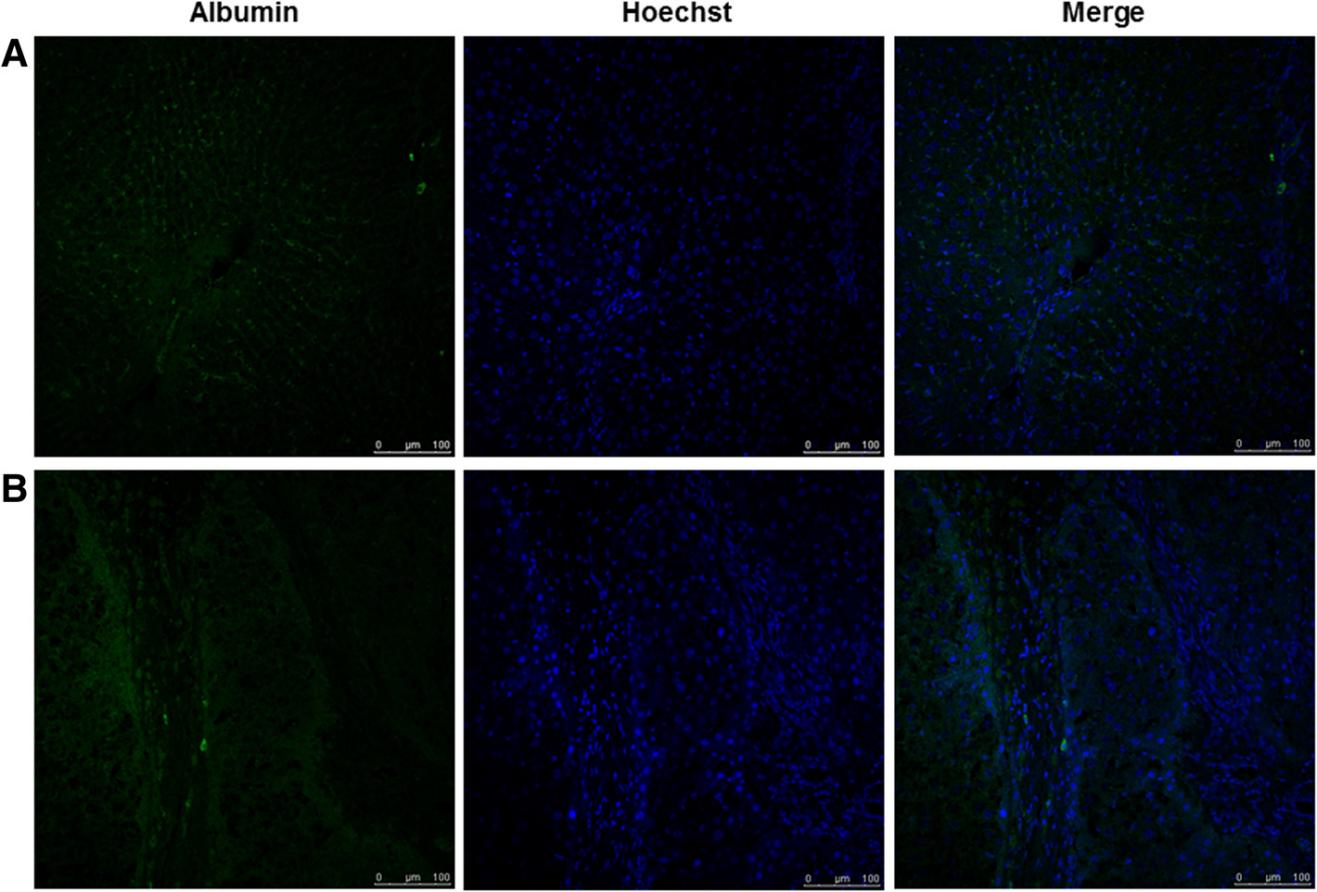
**Immunofluorescence staining for albumin expression after BM-MSCs injection by confocal microscope.** Immunofluorescence image showed BM-MSCs with hepatocyte differentiation and albumin expression at 7 days **(A)** and 14 days **(B)** after BM-MSCs injection under a confocal microscope. Merged immunofluorescence images of albumin (green) and hoechst (blue). (Scale bar, 100 μm).

To investigated whether BM-MSCs are capable of undergoing hepatic differentiation, the expression of albumin was detected by immunofluorescence staining. At 7 days after BM-MSCs injection, expression of albumin was weakly detected. However, intensity of albumin staining was stronger at 14 days (Figure 4).

To identify injected BM-MSCs to the hepatic fibrosis rat liver, fluorescence red labeled BM-MSCs were showed at 0, 3, 7, 14 days after direct injection in hepatic fibrosis rat liver. Fluorescence intensity from labeled BM-MSCs was maintained for 14 days in liver tissue. However fluorescence intensity from labeled BM-MSCs was gradually reduced and disappeared at 14 days (Additional file 1: Figure S1).

Differentiation of BM-MSCs into hepatocytes was determined by immunofluorescence staining using albumin antibody and identification of BM-MSCs in hepatic fibrosis was determined by fluorescent microscopy using CELL STALKER-CSR dye staining. Consequently, we showed that fluorescence intensity from labeled BM-MSCs with CELL-STALKER was gradually disappeared and albumin-stained areas had gradually expanded at 14 days after injection, considering that BM-MSCs had transdifferentiated into hepatocytes after injection. Based on these results, it may suggested that BM-MSCs had transdifferentiated into hepatocytes after injecting hepatic fibrosis rats.

### Biochemical analysis

**Figure 5:**
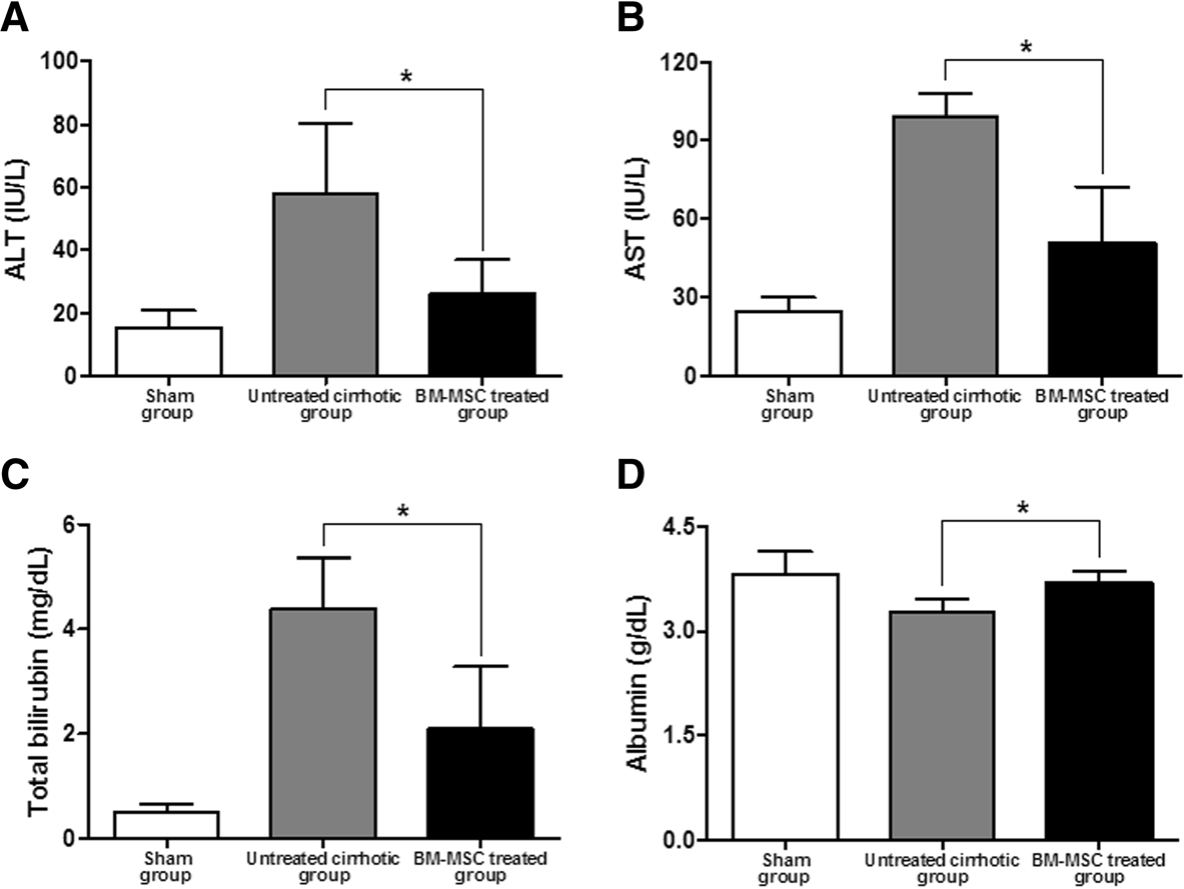
**Effect of BM-MSCs on serum biomarkers. (A)** Alanine aminotransferase (ALT), **(B)** aspartate aminotransferase (AST), **(C)** total bilirubin, and **(D)** albumin after injection of BM-MSCs. Values are expressed as means ± SD. *P <0.01.

The serum levels of ALT, AST, and total bilirubin were significantly decreased in BM-MSCs treated group. In addition, serum level of albumin in the BM-MSC treated group were increased compared to the untreated cirrhotic group (*P <*0.01; Figure 5).

### TGF-β1, collagen-1, and α-SMA mRNA levels

**Figure 6:**
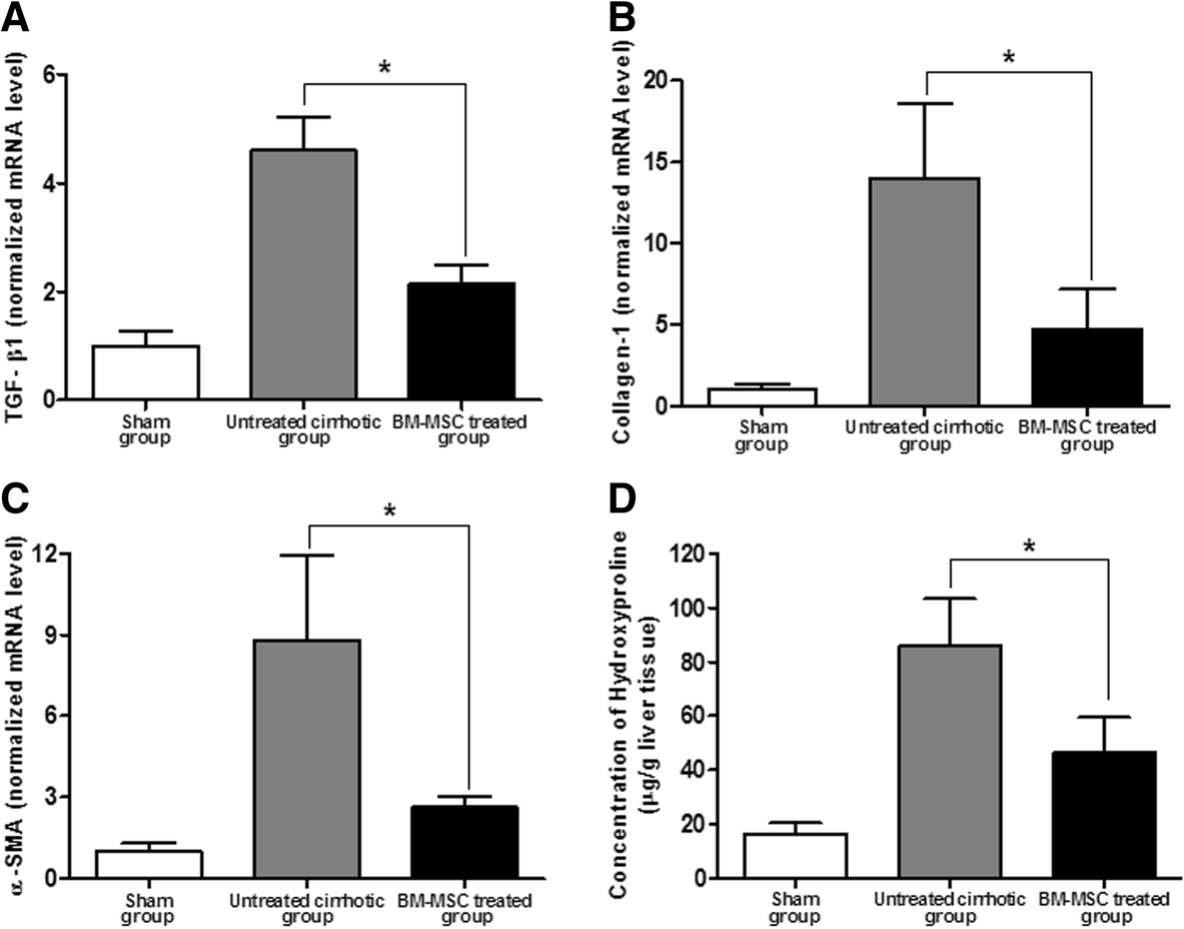
**TGF-β1, collagen-1, and α-SMA mRNA levels and measurement of hepatic hydroxyproline content.** After BM-MSC administration, the mRNA relative expression levels of TGF-β1 **(A)**, collagen-1 **(B)**, and α-SMA **(C)** through quantitative real-time PCR were significantly decreased 1 ± 0.27, 4.61 ± 0.61, and 2.14 ± 0.35 (*P <*0.01) and 1 ± 0.39, 14.04 ± 4.54, and 4.74 ± 2.46 (*P <*0.01) and 1 ± 0.32, 8.83 ± 3.12, and 2.62 ± 0.41 (*P <*0.01) in G1, G2, and G3, respectively. **(D)** Measurement of hepatic hydroxyproline content was quantified colorimetrically from liver tissues. Quantitative analysis showed that the contents of hepatic hydroxyproline of the liver tissue were 16.44 ± 4.07, 85.81 ± 17.62, and 46.25 ± 13.19 (*P <*0.01) in G1, G2, and G3, respectively. Values are expressed as means ± SD. *P <0.01.

After BM-MSC administration, the mRNA relative expression levels of TGF-β1, collagen-1, and α-SMA were determined through quantitative real-time reverse transcription-PCR (RT-PCR) analysis. The collagen-1 mRNA relative expression levels were 1 ± 0.39, 14.04 ± 4.54, and 4.74 ± 2.46 (*P* <0.01) in G1, G2, and G3, respectively (Figure 6B). In addition, the TGF-β1 and α-SMA mRNA relative expression levels were 1 ± 0.27, 4.61 ± 0.61, and 2.14 ± 0.35 (*P <*0.01) and 1 ± 0.32, 8.83 ± 3.12, and 2.62 ± 0.41 (*P <*0.01) in G1, G2, and G3, respectively (Figure 6A, 6C). The TGF-β1, collagen-1, and α-SMA mRNA relative expression levels in the BM-MSC treated group were significantly decreased compared to the untreated cirrhotic group (*P <*0.01).

### Measurement of hepatic hydroxyproline content

The content of hepatic hydroxyproline was quantified colorimetrically from liver tissues. Quantitative analysis showed that the contents of hepatic hydroxyproline of the liver tissue were 16.44 ± 4.07, 85.81 ± 17.62, and 46.25 ± 13.19 (*P <*0.01) in G1, G2, and G3, respectively (Figure 6D). The content of hepatic hydroxyproline was significantly lower in the BM-MSC treated group than in the untreated cirrhotic group.

### TGF-β1 and α-SMA protein expression levels

**Figure 7:**
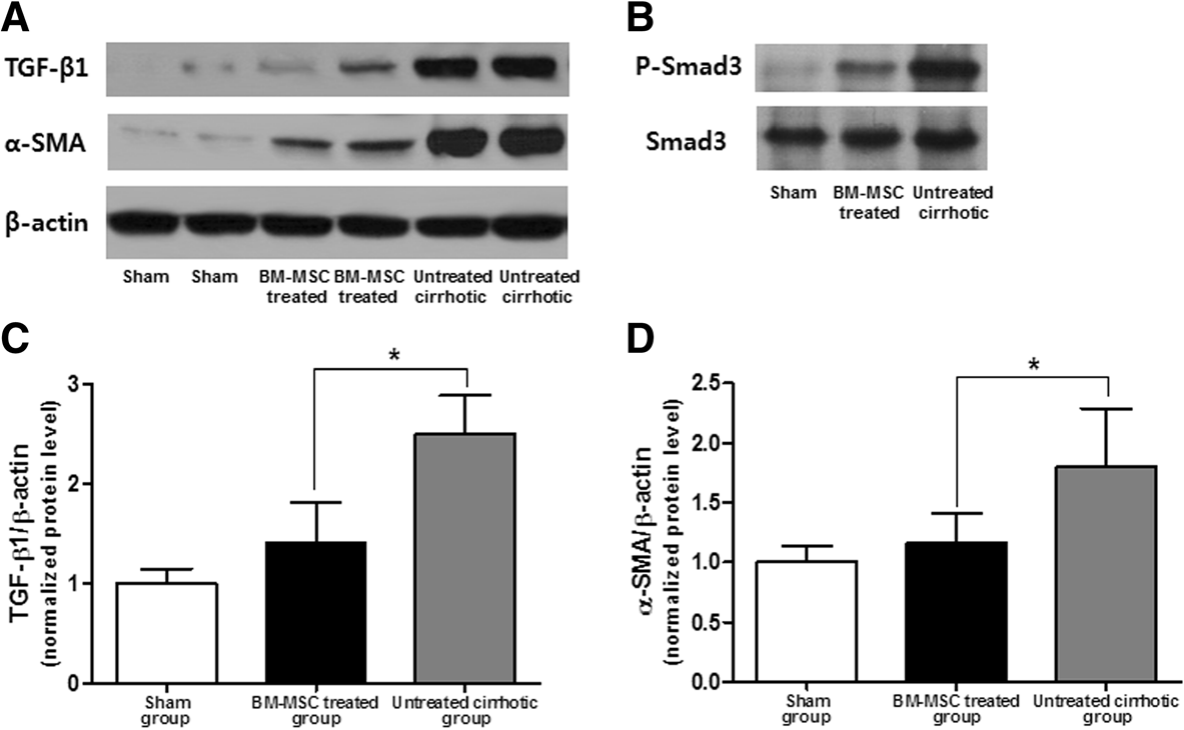
**TGF-β1, α-SMA, and Smad3 protein expression levels.** After BM-MSC administration, the TGF-β1 **(A, C)** and α-SMA **(A, D)** protein expression levels through western blot assays were significantly decreased 1 ± 0.15, 2.5 ± 0.39, and 1.41 ± 0.41 (*P <*0.01) and 1 ± 0.14, 1.8 ± 0.49, and 1.16 ± 0.25 (*P <*0.01) in G1, G2 and G3, respectively. **(B)** Phosphorylation of Smad3 was assessed through western blot assays. Values are expressed as means ± SD. *P <0.01.

The TGF-β1 and α-SMA protein expression levels were measured through western blot assays (Figure 7). The TGF-β1 and α-SMA protein expression levels were 1 ± 0.15, 2.5 ± 0.39, and 1.41 ± 0.41 (*P <*0.01) and 1 ± 0.14, 1.8 ± 0.49, and 1.16 ± 0.25 (*P <*0.01) in G1, G2, and G3, respectively (Figure 7). The TGF-β1 and α-SMA protein expression levels in the BM-MSC treated group were significantly decreased compared to the untreated cirrhotic group (*P <*0.01).

We examined the effect of BM-MSCs on the status of Smad3 and found that Smad3 phosphorylation was markedly increased in the untreated cirrhotic group but was significantly decreased after BM-MSC treatment. These results demonstrate that BM-MSCs modulated the TGF-β1/Smad signaling pathway by attenuating TGF-β1 and Smad3 expression and inhibiting Smad3 phosphorylation.

## Discussion

In this study, we investigated the effect of BM-MSCs on hepatic fibrosis in a TAA-induced cirrhotic rat model and explained the fundamental mechanism of hepatic fibrosis attenuation caused by BM-MSC treatment. The major findings of our study are as follows: (1) administration of MSCs at 2 × 10^6^ cells into TAA-induced cirrhotic rat livers via direct injection allowed recovery from TAA-induced fibrosis at 4 weeks after BM-MSC treatment; (2) BM-MSCs recovered liver function through decreases in TGF-β1, collagen-1, and α-SMA expression; and (3) BM-MSCs led to the recovery of liver function via the TGF-β1/Smad signaling pathway.

Recently, stem cell-based therapy has been proposed as a promising alternative approach for end-stage liver diseases. Stem cell therapies have shown promising benefits for hepatic fibrosis in experimental and clinical studies [11,12,17,20]. BM consists of two main populations of stem cells, hematopoietic stem cells and MSCs, the latter of which have been considered as alternative cell sources for liver or hepatocyte transplantation [22]. In liver damage, MSCs differentiate into hepatocytes, stimulate the regeneration of endogenous parenchymal cells, migrate to damaged sites, and enhance fibrous matrix degradation (antifibrotic effects). A recent study suggested that MSCs have antifibrotic effects on the injured liver in animal models of liver fibrosis [37]. Consistent with these results, we showed that the administration of BM-MSCs ameliorated hepatic fibrosis.

TAA-induced cirrhosis is a well-known classical experimental cirrhosis model [38,39]. In our study, we confirmed that TAA administration resulted in deficiencies in liver function and a progressive increase in collagen accumulation in the liver with periportal cirrhosis characterized by portal-portal fibrous septa surrounding the hepatic lobules.

Cirrhosis and advanced fibrosis are generally considered irreversible conditions, even after the removal of the hepatic injury. Through histological H&E and MTC, and α-SMA staining, we showed that BM-MSC administration resulted in significant improvement of hepatic fibrosis compared to the untreated cirrhotic group. There was a clear histological variability of severity within cirrhosis classified as F4 by the METAVIR system. Cirrhosis is currently considered potentially reversible if the cause of the injury is removed. The lack of subclassification within cirrhosis can be problematic when assessing the antifibrotic effect of agents such as antiviral drugs. For instance, even though antifibrotic therapy leads to the improvement of hepatic fibrosis from F4C to F4A in the Laennec system, the lack of change under the conventional METAVIR system may lead to the false conclusion that treatment is ineffective. Hence, further histological subclassification of cirrhosis is required [4,35]. In this study, we applied the new Laennec fibrosis scoring system to provide a more detailed classification of F4 cirrhosis. According to this scoring system, histological improvement was observed in hepatic fibrosis after BM-MSC treatment (*P* <0.01). These results were confirmed by immunohistochemical assays revealing α-SMA expression and Picrosirius Red staining. In addition, the percentage of the collagen proportionate area and the content of hepatic hydroxyproline significantly decreased after BM-MSC treatment. These results indicated that BM-MSCs improved liver structure in TAA-induced cirrhotic rats.

TGF-β1 is a primary mediator particularly in liver fibrogenesis. TGF-β1 promotes HSCs to transition into MFBs; it also stimulates the synthesis of ECM factors such as collagen-1 and inhibits its degradation [40]. Furthermore, the expression of α-SMA in the liver is an indicator of HSC activation, which is recognized as a key player in hepatic fibrosis and cirrhosis [41]. Likewise, this study showed that BM-MSCs recovered liver function, as indicated by decreased TGF-β1, collagen-1, and α-SMA gene expression. Furthermore, α-SMA protein expression significantly decreased after BM-MSC treatment. The results of our study are in agreement with those of Campbell JS et al. [42]. Indeed, the activation of TGF-β/Smad signaling is a key mechanism of liver fibrosis in both experimental and human chronic liver diseases [43]. We also showed P-Smad3/Smad3, downstream effectors of the TGF-β1 signaling pathway, and found that MSC transplantation inhibited Smad3 phosphorylation. Hence, this study has confirmed a significant inhibitory effect of BM-MSCs on TAA-induced cirrhosis in rats, which likely correlates with the TGF-β1/Smad signaling pathways.

## Conclusions

In conclusion, our study showed that BM-MSC treatment inhibited fibrosis formation and the progression of TAA-induced cirrhosis in rats by modulating the TGF-β/Smad signaling pathway. Therefore, BM-MSC therapy leads to the improvement of hepatic fibrosis and may provide a new strategy for antifibrosis therapy in the future.

## Additional file

Additional file 1: Figure S1. Fluorescence images of injected BM-MSCs labeled with CELL-STALKER in hepatic fibrosis rat. BM-MSCs labeled with fluorescence red were showed at 0 (A), 3 (B), 7 (C), 14 (D) days after direct injection in hepatic fibrosis rats (CELL STALKER-CSR dye staining, red: Scar bar, 100 μm).

## Abbreviations

BM: Bone marrow
BM-MSCs: Bone marrow-derived mesenchymal stem cells
MELD: Model for end-stage liver disease
TGF-β1: Transforming growth factor-beta 1
collagen-1: Type 1 collagen
α-SMA: α-smooth muscle actin
GAPDH: Glyceraldehyde-3-phosphate dehydrogenase
H&E: Hematoxylin and eosin
MTC: Masson’s trichrome
SD: Standard deviation
