# Supplementary figures and images for: Effect of bone marrow-derived mesenchymal stem cells on hepatic fibrosis in a thioacetamide-induced cirrhotic rat model
Source: BMC Gastroenterol. 2014 Nov 25;14:198. doi: 10.1186/s12876-014-0198-6 (PMC4251876; doi:10.1186/s12876-014-0198-6)

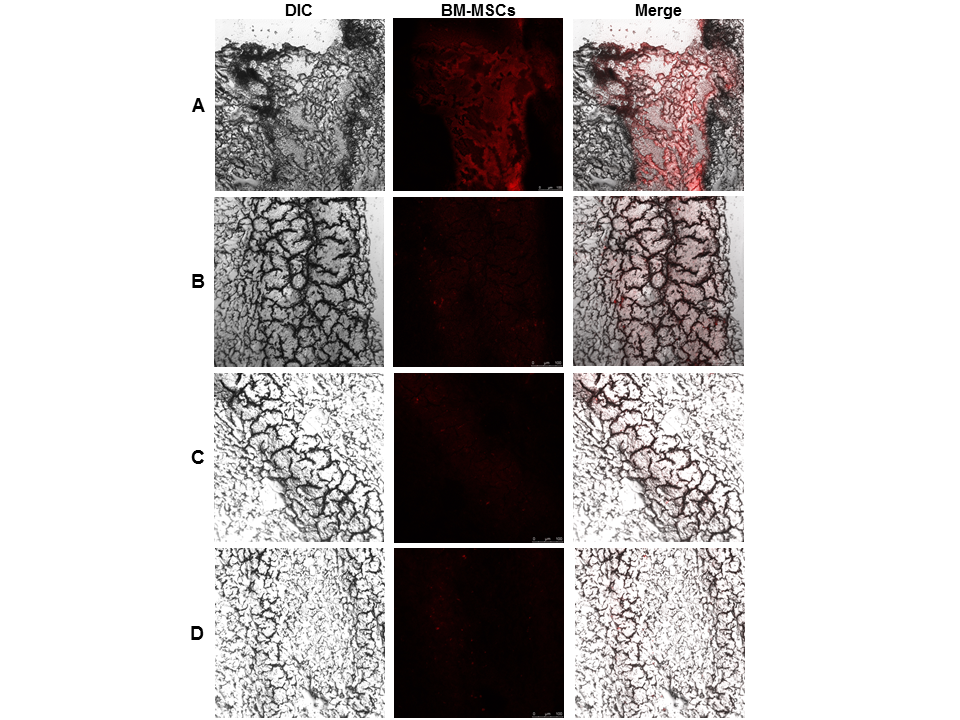

Supplement: Additional file 1: Figure S1. — Fluorescence images of injected BM-MSCs labeled with CELL-STALKER in hepatic fibrosis rat. BM-MSCs labeled with fluorescence red were showed at 0 (A), 3 (B), 7 (C), 14 (D) days after direct injection in hepatic fibrosis rats (CELL STALKER-CSR dye staining, red: Scar bar, 100 μm). [file 12876_2014_198_MOESM1_ESM.tiff]
